# Exploring the role of locomotor sensitization in the circadian food entrainment pathway

**DOI:** 10.1371/journal.pone.0174113

**Published:** 2017-03-16

**Authors:** Hanna Opiol, Nuria de Zavalia, Tara Delorme, Pavel Solis, Spencer Rutherford, Uri Shalev, Shimon Amir

**Affiliations:** Center for Studies in Behavioral Neurobiology, Department of Psychology, Concordia University, Montreal, QC, Canada; Kent State University, UNITED STATES

## Abstract

Food entrainment is the internal mechanism whereby the phase and period of circadian clock genes comes under the control of daily scheduled food availability. Food entrainment allows the body to efficiently realign the internal timing of behavioral and physiological functions such that they anticipate food intake. Food entrainment can occur with or without caloric restriction, as seen with daily schedules of restricted feeding (RF) or restricted treat (RT) that restrict food or treat intake to a single feeding time. However, the extent of clock gene control is more pronounced with caloric restriction, highlighting the role of energy balance in regulating clock genes. Recent studies have implicated dopamine (DA) to be involved in food entrainment and caloric restriction is known to affect dopaminergic pathways to enhance locomotor activity. Since food entrainment results in the development of a distinct behavioral component, called food anticipatory activity (FAA), we examined the role of locomotor sensitization (LS) in food entrainment by 1) observing whether amphetamine (AMPH) sensitization results in enhanced locomotor output of FAA and 2) measuring LS of circadian and non-circadian feeding paradigms to an acute injection of AMPH (AMPH cross-sensitization). Unexpectedly, AMPH sensitization did not show enhancement of FAA. On the contrary, LS did develop with sufficient exposure to RF. LS was present after 2 weeks of RF, but not after 1, 3 or 7 days into RF. When food was returned and rats regain their original body weight at 10–15 days post-RF, LS remained present. LS did not develop to RT, nor to feedings of a non-circadian schedule, e.g. variable restricted feeding (VRF) or variable RT (VRT). Further, when RF was timed to the dark period, LS was observed only when tested at night; RF timed to the light period resulted in LS that was present during day and night. Taken together our results show that LS develops with food entrainment to RF, an effect that is dependent on the chronicity and circadian phase of RF but independent of body weight. Given that LS involves reorganization of DA-regulated motor circuitry, our work provides indirect support for the role of DA in the food entrainment pathway of RF. The findings also suggest differences in neuronal pathways involved in LS from AMPH sensitization and LS from RF.

## Introduction

Circadian locomotor activity, predominantly under the control of the light/dark cycle, can be reorganized to adapt to daily time-restricted events of biological importance. Timekeeping mechanisms in the brain and/or body can anticipate a circadian event by shifting endogenous clock gene rhythms and behavioral locomotor output. Daily time-restricted access to a palatable treat [1, 2] or mating window [3, 4] has been shown to result in anticipatory behavioral activity prior to the scheduled event. Similarly, daily-timed injections of rewarding or DA-activating drugs show anticipatory activity preceding injection [5, 6]. The most notable example of circadian reorganization to a time-restricted event is the implementation of circadian RF. RF requires two components: 1) that complete caloric intake be restricted to a specific feeding window, usually a few hours in length and 2) that the timing of feeding does not vary extensively between each circadian cycle. The effects of RF can be seen as soon as the following day, including shifted clock gene expression, neuroendocrine signaling and the reorganization of circadian behavior [7]. The biological changes that arise from circadian scheduled feeding are referred to as food entrainment. The mechanism of food entrainment is unknown, however one of the defining behavioral features is the development of FAA prior to feeding time. Feeding-related behavior, including FAA, has been related to DA function in motor regions of the brain [6, 8]. Taken together, it is speculated that daily time-restricted events affecting DA function may alter components of the circadian clock, resulting in a mechanism by which organisms are able to anticipate the time of their occurrence.

One possibility is that clock genes within particular brain regions, such as those driving locomotor output, may be involved in the timekeeping of salient events by sensing changes to the circadian rhythmicity of DA. For example, stimuli that act on DA in a circadian fashion, when the organism deems the stimuli sufficiently salient (e.g. food under food-restricted conditions), may act as an input signal for clock genes regulating diurnal activity. DA has been shown to shift and regulate circadian locomotor output and influence clock genes in brain regions important for motor output [6, 9–15]. Of particular interest is the clock gene Per2, whose protein expression is under the control of DA in the dorsal striatum [11]. The role of Per2 in food entrainment remains undetermined [16], however mice with a mutation for Per2 show significant impairment of FAA [17], suggesting it is important for locomotor output of food entrainment. Interestingly, time has previously been shown to act as a conditioned stimulus for the locomotor expression of AMPH sensitization [18], and clock genes appear to play a critical role in the expression of behavioral sensitization [19–22]. Therefore, time-keeping mechanisms through which food entrainment reorganizes behavioral rhythms could involve neuroplastic changes to motor circuits that are also important for LS to reward-related stimuli [23].

The term LS refers to the enhancement in behavior due to the (repeated) application of a stimulus [24]. LS occurs as a result of long-term neurochemical changes in the brain, which produce hypersensitivity to neurotransmitter systems, including the DA system [25, 26]. One of the defining factors of LS is that the effects last beyond the cessation of whatever stimuli initiated the locomotor changes, and is often enhanced during the time of withdrawal or period of "incubation" [21]. Therefore, LS can be thought of as the composite of two critical stages: its development and its expression [27, 28]. Development involves neuroplastic changes that occur during the application of a repeated stimulus and during the incubation period after stimuli cessation. The expression of LS involves the persistence of the neuronal changes that occurred during development. Furthermore, when different stimuli act on the same neuronal networks involved in LS, often times cross-sensitization can be observed, such that LS resulting from one stimulus produces a hyper-response to the second stimulus [29]. A recent study found that the presence of a locked running wheel resulted in the enhancement of FAA in mice with previous running wheel exposure, while mice naive to a running wheel showed no change in FAA [30]. The authors hypothesized that FAA, like running wheel activity, involves reward-signaling mechanisms that can further be enhanced by rewarding stimuli.

In the first part of our study we tested whether behavioral properties of food entrainment (FAA) could be augmented by enhancing DA activity through repeated AMPH exposure (AMPH sensitization). Unexpectedly, we found that there were no additive effects of AMPH sensitization on FAA, both when testing between and within group designs. We wondered whether our control groups, which were also subjected to RF, could have developed LS as a result of caloric restriction [31–33]. The threshold-lowering effects of rewarding electrical brain stimulation by drugs of abuse that act on DA, such as AMPH, can be potentiated with caloric restriction, leading to the speculation that caloric restriction produces additive effects through the DA system [34, 35]. However, AMPH sensitization (repeated exposure) does not result in the augmentation of electrical brain self-stimulation [35], suggesting that sensitization of drug reward in the rewarding electrical brain stimulation paradigm and LS might be mediated by different neural mechanisms. Given the previous findings, we hypothesized that either AMPH sensitization does not act through the same neuronal networks as food entrainment, and therefore we should not expect to see additive effects in FAA. Or else, a second possibility might be that control groups that were subjected to RF, also developed LS, which would then result in a ceiling effect of FAA between groups. We further investigated the latter option.

The second part of our study evaluated whether RF could result in LS. We tested various circadian and non-circadian feeding schedules on its effects of cross-sensitization to AMPH. We found that the most important aspect of feeding-induced LS was the combination of a circadian feeding schedule and caloric restriction. Body weight appeared to be irrelevant for RF-induced LS. Previous work has shown that the effects of caloric restriction on the sensitization of drug reward are related to weight loss. The augmentation of drug reward by caloric restriction was reversed after one week of returning rats to free access feeding [36] and during longer periods of food restriction, the reversal of food-induced behavioral sensitization paralleled the return of body weight to baseline [35, 37, 38]. In our study rats were returned to free access feeding prior to testing, whereby they regained pre-RF body weight. Our findings suggest that it is the duration of RF and/or a period of incubation that is necessary for the behavioral expression of LS. These observations are consistent with the idea that endogenous mechanisms regulating food entrainment include LS.

## Methods and materials

### Subjects

One hundred fifty-eight male Wistar rats (Charles River, St. Constant) weighing between 125–200 grams at the beginning of experiment were individually housed in running wheel cages placed inside individual sound attenuating chambers. A 12:12 light/dark cycle was maintained for 2 weeks before beginning each experiment. Light intensity inside the cage measured ~300 lux. Rats had free access to tap water and rodent lab chow, except when subjected to scheduled feeding. Injections of AMPH or saline were administered to rats via intraperitoneal route. All experimental procedures were conducted under the guidelines of the Canadian Council of Animal Care and approved by the animal care ethics committee at Concordia University, Quebec.

### Feeding schedules

**Fig 1:**
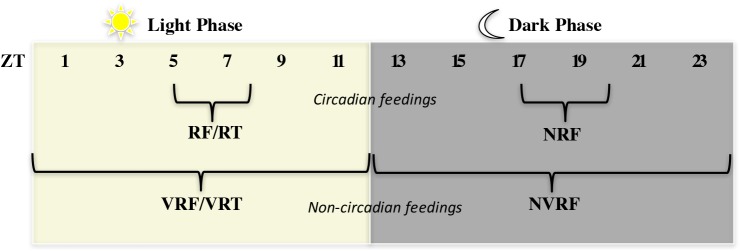
Schematic of daily feeding schedules. Circadian feedings occurred daily during a 2-3h feeding window during the lights on phase (RF/RT) or lights off phase (NRF). Non-circadian feedings occurred daily during a variable 2h-feeding window, restricted to the lights on phase (VRF and VRT) or lights off phase (NVRF).

Food (lab chow) was removed 22–24 hours prior to starting timed-restricted feedings with caloric restriction. As depicted in Fig 1, RF rats were fed during a daily 2-3h feeding window corresponding to Zeitgeber time (ZT) 5–8, where ZT 0 is lights on. Placing RF in the middle of the light period was done by convention to allow for a low background of activity from which FAA can be easily identified. Night RF (NRF) rats were fed during a daily 2-3h feeding window from ZT 17–20. To evaluate the effects of caloric restriction without food entrainment, variable feeding schedules were applied. Variable RF (VRF) and night VRF (NVRF) rats were fed at variable times each day between ZT 0–12 and ZT 12–24, respectively. Note that the hours for VRF were restricted to daytime as opposed to 24h-VRF due to a previous report of PER2 disruption in the dorsal striatum as a result of daytime-VRF but not 24h-VRF [42]. To evaluate the effects of food entrainment without caloric restriction, we allowed rats daily access to a restricted treat (RT) and variable RT (VRT). RT and VRT consisted of daily scheduled unlimited access to Oreo cookies (Cadbury, Mondelez-International) between ZT 5–7, and for 2 hours at variable times between ZT 0–12, respectively. RT and VRT had free access to lab chow at all times.

### Exp 1–3 protocols: AMPH sensitization effects on FAA

In experiment 1, rats were divided into 2 groups (*n* = 6) and given 5 x 1.5 mg/kg AMPH or saline every 48 hours during the lights on period ZT 4–6, followed by 7 days of incubation (no injection). Rats were then subjected to 12 days RF to evaluate FAA between groups.

In experiment 2, rats were subjected to RF for 2 weeks before AMPH sensitization (RF-before) followed by a 1-week break. Next rats were divided into 2 groups (*n* = 6) and given injections of AMPH, 3 mg/kg daily for 6 days (AMPH group) [39], or no injection (control group). Controls were not handled to minimize stress-induced sensitization that can occur from repeated saline injections [40]. AMPH injections occurred between ZT 14–16, 2-4h into the dark period to mimic the natural rhythm of DA in the dorsal striatum [11]. A period of incubation (10 days) followed AMPH sensitization and rats were subjected to RF a second time (RF-after) for 2 weeks. Rats were again returned to free access feeding for 1 week and subjected to an AMPH challenge test to see whether differences in LS between AMPH pretreated rats and controls was present. Since non-handled controls used in this protocol had not previously been subjected to injections, a habituation day was done on the day prior to the AMPH challenge. On habituation day both groups received an injection of saline and open-field activity was measured. On AMPH challenge day both groups received a low dose of AMPH (.5 mg/kg) and open-field activity was measured again. Challenge injections occurred between ZT 2–8.

**Fig 2:**
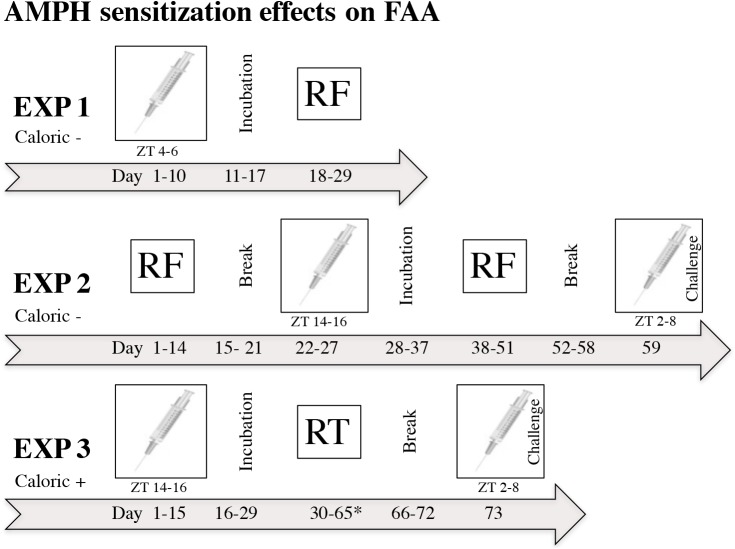
Exps 1–3: protocols for evaluating effects of AMPH sensitization on FAA. Experiments 1 and 3, use a between groups design to evaluate FAA in AMPH pretreated rats and controls, and experiment 2 uses a within group design to evaluate FAA before and after AMPH sensitization. Caloric restriction (-) or surplus (+) indicates the use of RF or RT, respectively, in each experiment. Timelines for AMPH sensitization and scheduled feeding are described below. Exp 1: rats were subjected to AMPH sensitization followed by an incubation period and RF. Exp 2: rats were placed on RF before AMPH sensitization (RF-before), followed by a break, and then divided into 2 groups: non-handled control or AMPH for AMPH sensitization, followed again by a break (incubation) and RF (RF-after). Rats were next returned to free access feeding and then challenged with a low dose of AMPH to see whether differences in LS were present between AMPH-pretreated rats and controls subjected to RF. The day prior to the AMPH challenge (day 58), a habituation run using a saline injection was conducted. Exp 3: rats were divided into 2 groups and subjected to AMPH sensitization followed by an incubation period prior to being placed on RT for 5 weeks. *During week 5 (days 59–65) the lights remained off (DD). As in experiment 2, following a break rats were challenged with AMPH to see whether differences in LS were present between AMPH pretreated rats and controls subjected to circadian feeding with no caloric restriction (RT). Both AMPH challenge tests (Exp 2 and 3) occurred during the lights on phase between ZT 2–8, consistent with time of previous FAA expression. Each AMPH sensitization protocol was selected based on the criteria that a prior study verified it to result in an increase of extracellular DA in the striatum and/or enhanced locomotor activity.

In experiment 3, rats were divided into 2 groups (*n* = 6) and given 5 x 1.5 mg/kg AMPH or saline every 72 hours, during ZT 14–16, followed by 14 days incubation [41]. Rats were then subjected to 5 weeks of RT. To observe whether there were any masking effects of light on FAA, week 5 of RT was conducted in constant darkness. Following the cessation of RT, rats underwent a 1-week break and were subjected to an AMPH challenge. For the challenge test, both groups received a low dose of AMPH between ZT 2–8 and open-field activity was measured. See Fig 2 for a timeline of AMPH sensitization protocols in relation to feeding schedule used in experiments 1–3, to investigate the effects of AMPH sensitization on FAA.

### Exp 4–9 protocols: Feeding cross-sensitization to AMPH

In experiments 4–8, rats were divided into groups determined by feeding schedule (RF, VRF, RT, VRT, NRF or NVRF). Control (CTRL) groups had no changes to diet. Feeding schedules were followed by 10–15 days of free access (ad libitum, AL) feeding, which served as time for rats to regain body weight and as an incubation period. After the incubation period all rats were administered a low dose of AMPH (.5 mg/kg) to test for cross-sensitization of locomotor activity in an open-field box. Groups were assigned as follows. Exp 4: rats were divided into 2 groups, CTRL or RF (*n* = 6), that were further subdivided to include a saline injection subgroup for the cross-sensitization test. Exp 5: rats were divided into 3 groups: CTRL, RF or VRF (*n* = 4). Exp 6: rats were divided into 3 groups: CTRL, RT, and VRT (*n* = 4). Exp 7–8: rats were divided into 5 groups: CTRL, RF, VRF, NRF and NVRF (*n* = 5). Injections for the cross-sensitization test occurred during the light phase (ZT 2–8), with the exception of experiment 8, where injections occurred during the dark phase (ZT 14–17).

**Fig 3:**
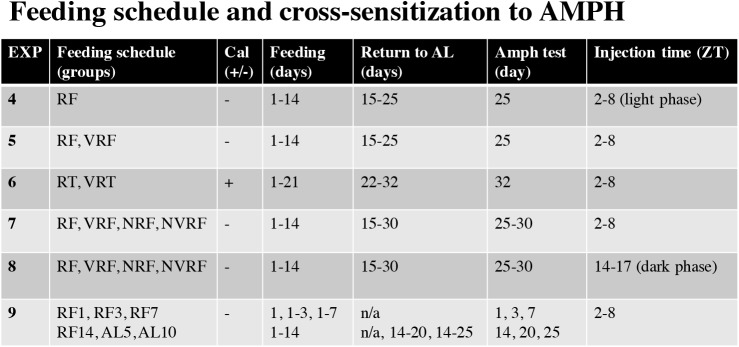
Exps 4–9: protocol timelines for testing feeding schedule cross-sensitization to AMPH. In experiments 4–8, different feeding schedules were tested for cross-sensitization to AMPH 10–15 days after returning to free access feeding (ad libitum, AL). In experiment 9, cross-sensitization of RF was tested on days 1, 3, 7 or 14 of RF (RF1, RF3, RF7, RF14) and at 5 or 10 into ad libitum feeding following 2 weeks of RF (AL5, AL10). The use of caloric (cal) restriction or surplus by experimental design are indicated with—or +, respectively. CTRL rats had no changes to diet and were tested on the same day as feeding groups. Experiment 4 also included a saline injection subgroup as a second control group on AMPH test day. All injections occurred between ZT 2–8, the time of day corresponding to FAA in RF rats, with the exception of experiment 8 where injections occurred between ZT 14–17, corresponding to FAA for NRF rats. Feeding group abbreviations: RF = restricted feeding, VRF = variable RF, RT = restricted treat, VRT = variable RT, NRF = night RF, NVRF = night VRF, CTRL = control.

In experiment 9, rats were divided into 7 groups (RF1, RF3, RF7, RF14, AL5, AL10 and CTRL), RF1, RF3, RF7 and RF14 were subjected to RF for 1, 3, 7 and 14 days, respectively, and tested for cross-sensitization on the last day of RF. AL5 and AL10 were subjected to 14 days RF and tested for cross-sensitization at 5 or 10 days post-RF. CTRL remained on free access feeding and were evenly divided across days of AMPH cross-sensitization tests. Injections for the cross-sensitization test occurred during the light phase (ZT 2–8). See Fig 3 for a detailed timeline of cross-sensitization protocols in experiments 4–9.

### Data collection and analysis

Running wheel activity was collected from the home cage of each rat using VitalView software (Mini Mitter, Bend, OR) and recorded in 10 min bins. The data was used to graph activity as group averages during different stages of each experiment. FAA ratios were calculated using the sum running activity 3h prior to feeding (ZT 2–5) divided by the sum nocturnal running activity (ZT 12–24).

Open-field data was collected using Tru Scan 2.0 activity monitoring system (Coulbourn Instruments, Whitehall, PA). Three or four rats were tested simultaneously within the same room using separate boxes (39 x 42 x 50 cm) with plexiglass transparent walls placed inside sound attenuation boxes constructed from insulation board. Total distance covered (cm) was collected in 5 min bins during a period of 1.5 h (30 min habituation, followed by 60 min post-injection). Distance-travelled ratios were later calculated as a measure of LS by summing distance travelled post-injection divided by total distance travelled.

Raw body weight scores were recorded for each group across stages of the experiment. To check whether rats had regained their pre-scheduled feeding (pre-RF/RT) body weight by test day, the percent pre-RF/RT weight was calculated (using weight on test day and pre-RF/RT weight).

### Statistical analysis

Two-way repeated measures ANOVA was used to evaluate FAA ratios, with day or week of RF as the within subject time factor and AMPH sensitization versus control as the between subject group factor. Unpaired t-tests, one-way ANOVA or two-way ANOVA (in the case that injection was a factor itself) were used to evaluate open-field behavior by comparing the group means of distance-travelled ratios or total distance travelled post-injection. Post-hoc analyses, indicated below, were applied when the test yielded significance. Rats with ineffective injections were excluded from analysis.

## Results

### Exp 1–3: AMPH-pretreated rats do not show enhancement of FAA to RF or RT

Experiments 1–3 examined whether behavioral response of FAA is enhanced as a result of AMPH sensitization, since AMPH sensitization enhances DA activity in the dorsal striatum [43] and the dorsal striatum has been implicated in food entrainment [6]. We hypothesized that if food entrainment acts through the same DA signaling pathway as AMPH sensitization, then there may be additive effects in the form of increased FAA in AMPH-pretreated rats subjected to a circadian feeding schedule.

**Fig 4:**
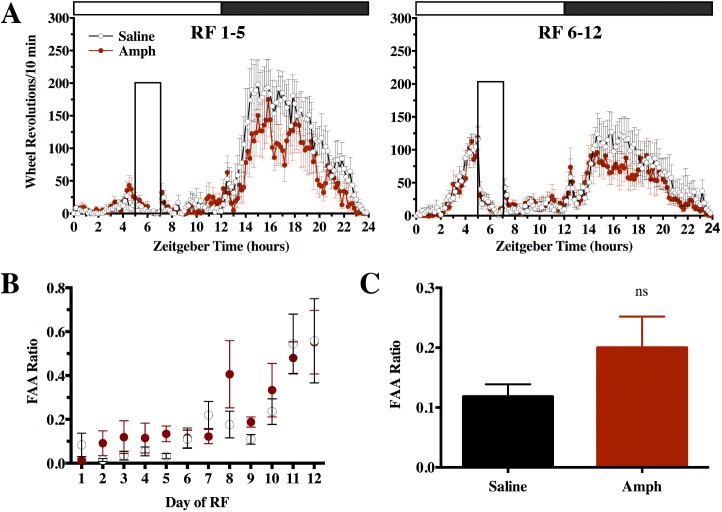
Exp 1: measuring FAA to RF in rats previously exposed to AMPH or saline injections, between group design. Home-cage activity expressed in wheel revolutions /10 min +/- SEM for AMPH and saline pretreated rats, during RF days 1–5 and 6–12, closed box indicates feeding window (**A**). FAA ratios +/- SEM shown as group averages for each day of RF (**B**) and for the total 12 days of RF (**C**), not significant, *ns*.

In experiment 1, AMPH-pretreated rats and saline controls were placed on RF to see whether there would be any differences in FAA as measured by FAA ratios. Fig 4A shows FAA development for rats pretreated with saline or AMPH. Both groups increased FAA from day 1 to 12 as expected, *F*_(11, 110)_ = 11.99, *p* < 0.01, but there were no differences between groups *F*_(1, 10)_ = 0.48, *p* = 0.51 and there was no interaction between time and group, *F*_(11, 110)_ = 0.84, *p* = 0.60 (Fig 4B). FAA ratios averaged across total RF time also did not differ significantly, *t*_10_ = 1.47, *p* = 0.17 (Fig 4C).

**Fig 5:**
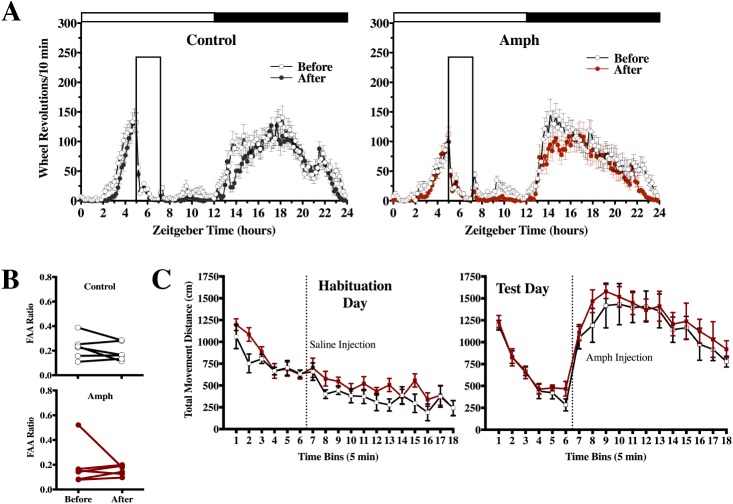
Exp 2: measuring FAA to RF before and after AMPH sensitization compared to non-handled controls, within group design. Home-cage activity expressed in wheel revolutions /10 min +/- SEM before and after AMPH sensitization for AMPH-pretreated and non-handled controls, closed box indicates feeding window (**A**). FAA ratios +/- SEM expressed as individual rat FAA ratios during RF-before and RF-after (**B)**. AMPH-pretreated rats and non-handled controls injected with saline on habituation day and AMPH on test day, with open-field activity measured as total movement distance +/-SEM in 5 min bins (**C**).

In experiment 2, rats were tested for within group differences in FAA before and after AMPH treatment (Fig 5A). Fig 5B shows no differences in FAA when comparing RF-before to RF-after in rats injected with AMPH and non-handled controls as measured by FAA ratios; time factor *F*_(1,10)_ = 1.14, *p* = 0.31, group factor *F*_(1,10)_ = 0.52, *p* = 0.49 and no interaction *F*_(1,10)_ = 0.002, *p* = 0.96. Unexpectedly, at the end of the experiment when all rats underwent an AMPH challenge procedure, there was no difference in distance-travelled ratios between groups, both on habituation day and on the following AMPH challenge day (*t*_10_ = 0.94, *p* = 0.37 and *t*_10_ = 0.32, *p* = 0.76, respectively). The results suggested that RF could have resulted in locomotor sensitization of the control group (Fig 5C).

**Fig 6:**
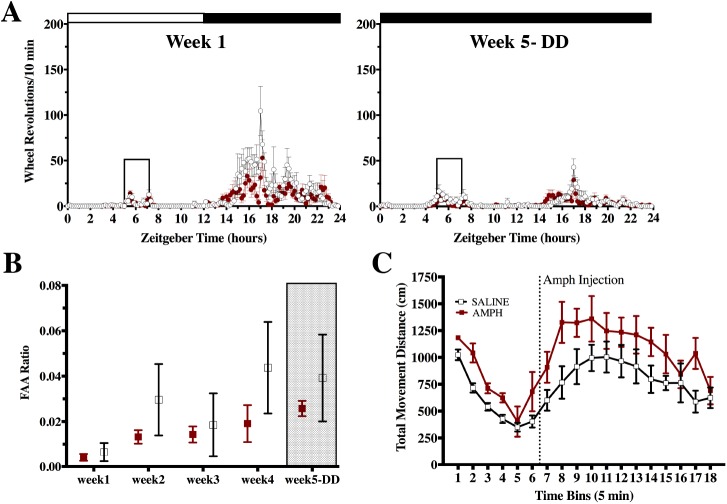
Exp 3: measuring FAA to a RT in rats previously exposed to AMPH or saline injections, between group design. Home-cage activity of AMPH and saline pretreated rats subjected to RT during week 1 and week 5 (constant darkness, DD), expressed in wheel revolutions /10 min +/- SEM, closed box indicates feeding window (**A**). FAA ratios +/- SEM for each week of RT (**B**). Open-field activity measured as total movement distance +/- SEM in 5 min bins for AMPH and saline pretreated rats injected with AMPH (**C**).

In experiment 3, we tested whether rats fed a daily RT, without caloric restriction, would show enhancement of FAA when pretreated with AMPH compared to saline controls. RT is known to induce FAA prior to a daily scheduled treat, although this activity is not as robust as FAA in rats with caloric restriction and takes longer to develop [1, 2]. Even by prolonging the feeding schedule of RT and placing rats into constant darkness to check for any masking effects of light on activity during the final week, we found no difference in FAA between rats pretreated with saline or AMPH as measured by FAA ratios, *F*_(1, 10)_ = 0.85, *p* = 0.38. Both groups increased FAA from week 1 to week 5, *F*_(4, 40)_ = 4.40, *p* < 0.01, with no significant interaction between time and group *F*_(4, 40)_ = 0.73, *p* = 0.58 (Fig 6A and 6B). At the end of the experiment, an AMPH challenge was conducted, which appeared to show that AMPH-pretreated rats were more active than controls (Fig 6C), although these distance-travelled ratios were not significantly different *t*_9_ = 0.31, *p* = 0.76, and neither were scores summing total activity post-injection, *t*_9_ = 1.69, *p* = 0.13.

### Exp 4–9: Crossover LS of circadian feeding to AMPH depends on caloric restriction, time of day and continuity of circadian RF

Experiments 4–9 tested the hypothesis that RF itself resulted in LS. We suspected that the reason experiments 1–3 failed to show an enhancement in FAA after AMPH treatment was that RF resulted in LS of the control group, which could then produce a ceiling effect in FAA between groups. We reasoned that if circadian RF resulted in sensitization of motor output, then possibly it was through the same mechanism as AMPH sensitization and therefore crossover sensitization should be possible from RF to an acute injection of AMPH. To test this hypothesis we placed rats under various feeding paradigms, returned them to AL feeding to restore body weight and then administered a low-dose of AMPH to measure locomotor activity in an open-field test. Feeding schedules varied, with the intent of testing the importance of caloric versus non-caloric restriction, circadian versus non-circadian feeding times, and feeding windows restricted to the day versus night. Because experiments 4–8 tested for cross-sensitization after a period of incubation (10–15 days post-RF), in experiment 9 we tested cross-sensitization during RF after 1, 3, 7 and 14 days of RF as well as 5 and 10 days post-RF.

**Fig 7:**
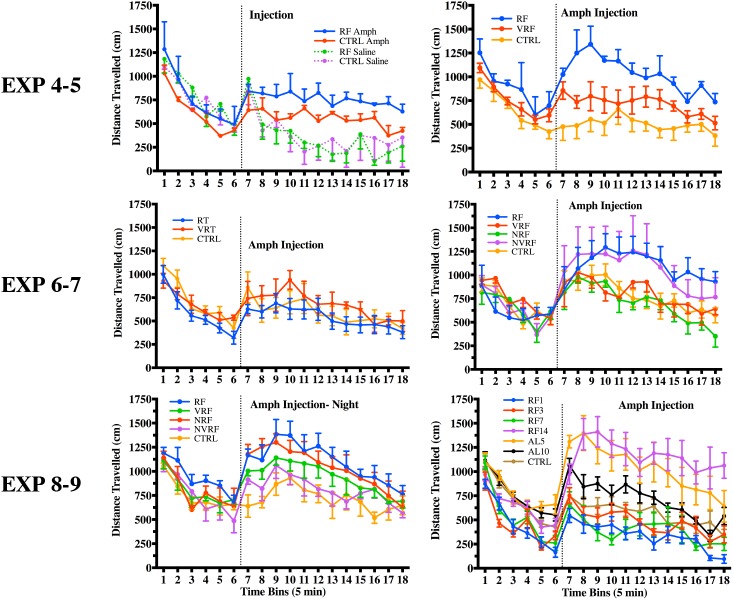
Feeding schedule cross-sensitization to AMPH test. Distance travelled to an acute injection of AMPH, plotted in 5-min time bins +/- SEM for feeding groups in experiments 4–9, respectively. Dotted line indicates injection time.

In experiment 4 we tested whether RF resulted in cross-sensitization to AMPH 10 days post-RF. On test day, feeding groups were divided and administered an acute injection of AMPH or saline. A significant difference in distance travelled scores was found between injection treatment groups *F*_(1, 8)_ = 15.35, *p* < .01, but not between feeding groups, *F*_(1, 8)_ = 0.08, *p* = 0.78, and there was no interaction, *F*_(1, 8)_ = 0.17, *p* = 0.69. Sidak's multiple comparison found differences between RF-AMPH and RF-saline, but not between CTRL-AMPH and CTRL-saline (Fig 7).

In experiment 5, we tested whether cross-sensitization to AMPH was present in rats fed a VRF schedule. On test day differences in open-field activity between groups was detected, *F*_(2, 8)_ = 4.62, *p* < 0.05. Dunnett's post hoc test (α < .05) revealed RF to be significantly higher than CTRL, but not VRF compared to CTRL (Fig 7).

In experiment 6, we tested whether cross-sensitization to AMPH was present in rats subjected to RT or VRT. On test day there were no statistically significant differences in open-field activity between RT and VRT compared to CTRL, *F*_(2, 9)_ = 1.91, *p* = 0.20 (Fig 7).

In experiment 7, we tested whether cross-sensitization to AMPH was present in rats subjected to RF, VRF, NRF and NVRF and found that there was a significant increase in open-field activity between groups, *F*_(4, 18)_ = 2.98, *p* = 0.05. Dunnett's multiple comparison (α < .1) revealed RF to be significantly higher than CTRL. NVRF, although it appeared to have a high response to AMPH, was not statistically significantly different than CTRL (Fig 7). Upon inspection of individual rats’ responses for NVRF, we found high variability that was evenly divided; 2 rats had high responses and 2 rats had a response similar to CTRL.

In experiment 8, we tested whether cross-sensitization was present in rats subjected to RF, VRF, NRF and NVRF when tested during the lights off phase. On test day there was a significant difference in open-field activity between groups, *F*_(4, 16)_ = 3.35, *p* = 0.04. Dunnett's multiple comparison (α < .1) revealed differences for RF and NRF compared to CTRL (Fig 7).

In experiment 9, we tested whether cross-sensitization to AMPH was present in rats during day 1, 3, 7 or 14 of RF (RF1, RF3, RF7, RF14) and 5 or 10 days post 2 weeks RF (AL5, AL10) compared to CTRL rats. A significant difference in open-field activity was detected between groups, *F*_(6, 37)_ = 7.607, *p* < 0.01. Dunnett's revealed significant differences for RF14 and AL5 compared to CTRL (Fig 7).

#### Percent of pre-RF/RT body weight on cross-sensitization test day

Rats typically regained 100% pre-RF/RT body weight by test day, indicating RF cross-sensitization to AMPH does not run in parallel to the return of body weight (S1 Fig).

## Discussion

Restricting caloric intake is known to enhance behavioral response to drugs of abuse in rats. We thought to investigate whether the opposite could be true of circadian RF, namely whether enhancing LS through repeated AMPH exposure would enhance FAA to RF. The latter hypothesis was of particular interest since AMPH sensitization is known to act through the DA system and food entrainment is believed to involve DA. Therefore, if both AMPH sensitization and food entrainment act through the same DA neuronal networks, then we should see additive effects on FAA. We found no evidence for AMPH sensitization enhancing FAA. This outcome was not completely explained by our second hypothesis that RF resulted in LS of control groups and therefore a ceiling effect in FAA, when compared to AMPH-pretreated rats subjected to RF.

With the second part of our study we verified that RF results in LS as measured by RF cross-sensitization to an acute injection of AMPH. LS from RF was found to require more than one week for behavioral expression, since an acute injection of AMPH on days 1, 3, and 7 of RF did not result in cross-sensitization. The latter finding was unexpected, as we had expected LS to be present as soon as caloric restriction was implemented. Therefore, if LS does not occur during the initial stages of food entrainment, some alternative explanation needs to be offered for why FAA among control groups did not differ from AMPH pretreated rats during the first week of RF in experiments 1–3. One explanation may be related to the high variability and instability of FAA during the initial stages of food entrainment, typically seen in the first week of RF. Although a more plausible explanation is that AMPH sensitization and food entrainment have partly different neuronal mechanisms that do not overlap to produce additive effects on downstream locomotor output. In the intracranial self-stimulation paradigm it has been shown that caloric restriction increases AMPH reward, while AMPH sensitization does not increase AMPH reward [35]. Our results may likewise be attributed to the inability of AMPH sensitization to produce additive effects on FAA.

The effectiveness of the chosen AMPH sensitization protocols to our experimental design may also be questioned. It is possible that AMPH sensitization protocols were not ideal for home-cage testing. Placing animals into a novel environment following AMPH injection facilitates sensitization [44] something that we did not do in the current protocols; however, the doses used for each protocol were high enough for sensitization to occur regardless of environmental conditions [45]. Different doses of AMPH and diurnal time of administration during sensitization and incubation have also been shown to produce differences in behavioral sensitization [46, 47] and these factors were taken into account when selecting AMPH sensitization protocols. Additionally, AMPH withdrawal is known to result in an initial decrease in DA levels in the dorsal striatum [40], which is believed to contribute to the sensitization process. Since we did not measure DA levels directly, we cannot confirm whether DA levels in AMPH-pretreated rats were low at initiation of RF, and perhaps this was why we did not observe FAA enhancement.

Given that DA is involved in food entrainment [6, 12, 48, 49], a second reason why FAA may not have increased in rats repeatedly exposed to AMPH is that the neuroplasticity of DA through which AMPH achieves behavioral sensitization is different from the changes in DA that occur with RF. For example, AMPH sensitization relates more to dopaminergic changes involving the D1 receptor, rather than D2 [41, 50, 51]. Circadian links to DA appear to work closely with D2 receptor function, e.g. clock gene expression in the dorsal striatum [11] and FAA is shifted by treatment with D2 receptor agonists, but not D1 agonists [12] or D1 antagonists [52]. Future experiments should test whether there are additive effects on FAA with psychostimulants that achieve behavioral sensitization through the DA D2 receptor [53, 54].

Since rats also show FAA to a RT, we examined whether a circadian feeding schedule without caloric restriction would show differences in FAA after being pretreated with AMPH. No differences in FAA were observed in AMPH-pretreated rats compared to controls while on a RT schedule, despite maintaining the schedule for an extended time and placing rats into constant darkness to unmask potentially hidden locomotor activity. Additionally, both RT and VRT schedules did not result in crossover sensitization to an acute injection of AMPH, indicating that some element of negative energy balance may be necessary for feeding-related LS. Circadian feeding schedules without caloric restriction do not alter forebrain and hypothalamic oscillators to the same extent as RF [55, 56]. Caloric restriction is an important aspect contributing to the clock function of food entrainment and likewise is necessary for circadian feeding-induced LS.

As seen in experiments 4–8, cross-sensitization to AMPH in groups subjected to circadian RF was present when tested 10–15 days post-feeding. Experiment 9 showed that RF did not result in LS during the first week of RF. Similar findings have been demonstrated with acute food deprivation, and its failure to enhance brain stimulation reward [57]. FAA also requires about a week before it reaches its maximum and stable amplitude in daily locomotor activity, suggesting that LS may be related to the enhancement (or reorganization) of locomotor activity during the first week of RF. One limitation of our study is that we cannot rule out the possibility that behavioral expression of LS may have been detected earlier had we used a higher dose of AMPH. Whether the expression of LS can be present during the first week of RF with a higher AMPH dose, or whether brain neuroplastic changes that underlie LS require more than a week to manifest as behavioral enhancement remains to be determined. It does not appear that LS is related to the amount of daily running wheel activity between groups. Both RF and VRF groups have similar levels of 24-h running activity, which are divided equally between the night and day (S2 Fig). LS, if affected by locomotor activity, may be related to the way it is reorganized across the 24-h cycle during the period of RF rather than the total amount of activity itself.

LS in RF but not VRF may be explained by RF's ability to maintain shifted clock gene expression. Recent studies have implicated transcription factors that comprise the molecular circadian clock to changes of neural plasticity [23]. By maintaining the same feeding time, RF may result in more consolidated neuroplastic changes important for LS, whereas VRF, which must readjust clock gene expression on a daily basis [58], may not. It is important to note that even though changes to clock gene expression by VRF may not be to the same extent as RF, VRF has been shown to dampen rhythms of PER2 in several brain regions, including the dorsal striatum [59], which may be relevant to the development or maintenance of RF LS. Finally, since we did not test whether LS is present in VRF groups during the VRF period, we cannot say that caloric restriction during VRF would not result in cross-sensitization; only that LS is maintained 10–15 days post-RF but not post-VRF. Therefore, LS may persist longer after cessation of circadian RF than non-circadian caloric restriction.

It is uncertain why cross-sensitization to AMPH was present in some NVRF rats when tested during the day, as this response was not present when NVRF were tested at night. In contrast, rats fed a circadian feeding schedule at night (NRF) showed cross-sensitization only when tested during the night. Unpredictable schedules involving non-caloric/ non-circadian food rewards have also been shown to result in cross-sensitization to a low dose of AMPH [60]. Both in the latter and present study the effects of cross-sensitization were unrelated to body weight loss. Similarly, Avena & Hoebel evaluated various feeding schedules comprised of restricted access to sugar and/or lab chow and came to the hypothesis that cyclic feeding affects DA signaling to produce sensitization independently of body weight changes [29].

Our results show that the combination of circadian feeding and caloric restriction result in LS as measured by an acute injection of AMPH. VRF, RT and VRT do not result in LS and NVRF shows high within-group variability. Interestingly, NRF only resulted in cross-sensitization when AMPH was administered during the night, while daytime RF resulted in cross-sensitization irrespective of the time of day. The latter may not be surprising, given that daytime RF results in the uncoupling of circadian oscillators from the SCN, such that both brain and peripheral oscillators can oscillate in anti-phase compared to oscillations under free-feeding conditions [61]. The amount of time it takes for non-SCN oscillators to resynchronize to the SCN, once animals are returned to free-feeding conditions, has not been extensively studied; however it is reasonable to hypothesize that brain regions important for both LS and/or food entrainment may remain coupled to the timing of feeding for some extended time post-RF. Unexpectedly, LS was not present during the first week of RF, but was present at 2 weeks into RF and 10–15 days post-RF. Body weight per se did not appear to play a role in these results since rats typically regained pre-RF body weight by test day. These results signify that food entrainment can play a role in experimental outcomes when chronic food restriction is used to study drug addiction. Moreover, LS resulting from circadian RF appears to be a distinguishing feature of food entrainment, and further supports the role of DA in the food entrainment pathway.

## Supporting information

S1 Fig Percent pre-RF/RT body weight +/- SD for individual rats within each group for experiments 4–9, respectively. (TIF)

S2 Fig Running wheel activity by stage of experiment. To see whether the total amount of locomotor activity across stages of the experiment differed between feeding groups, total daily wheel revolutions for each rat were summed and averaged for each stage of the experiment, in Exp's 4–8. Group averages of total daily wheel revolutions (+/- SEM) show the amount of activity that occurred in the day, night or over 24-h.
(TIF)

S3 Fig Feeding schedule cross-sensitization to AMPH test: Individual rats. Distance travelled to an acute injection of AMPH, plotted in 5-min time bins +/- SEM for feeding groups in experiments 4–9, respectively. Dotted line indicates injection time.
(TIF)
