# Supplementary figures and images for: Exploring the role of locomotor sensitization in the circadian food entrainment pathway
Source: PLoS One. 2017 Mar 16;12(3):e0174113. doi: 10.1371/journal.pone.0174113 (PMC5354457; doi:10.1371/journal.pone.0174113)

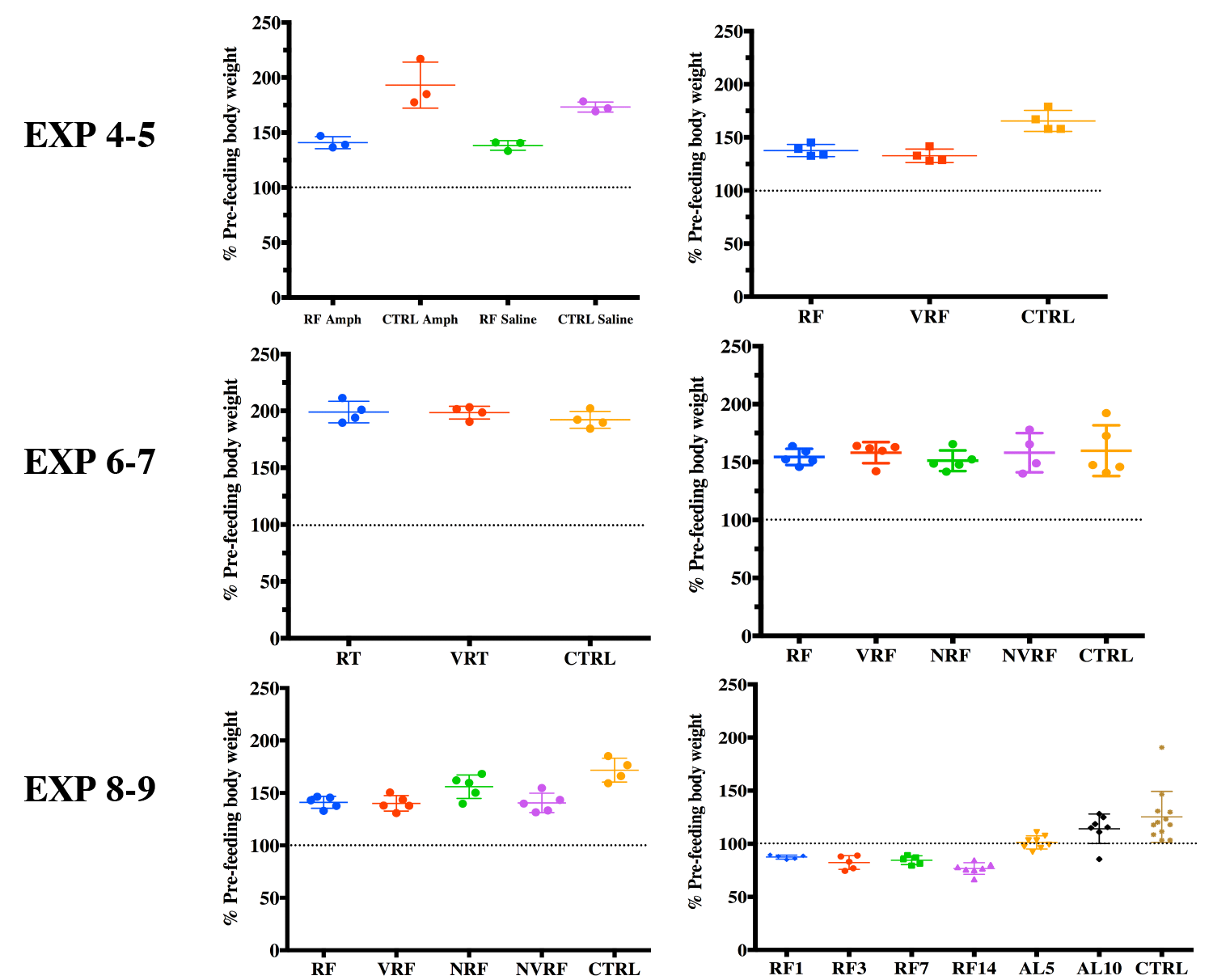

Supplement: S1 Fig — (TIF) [file pone.0174113.s001.tif]

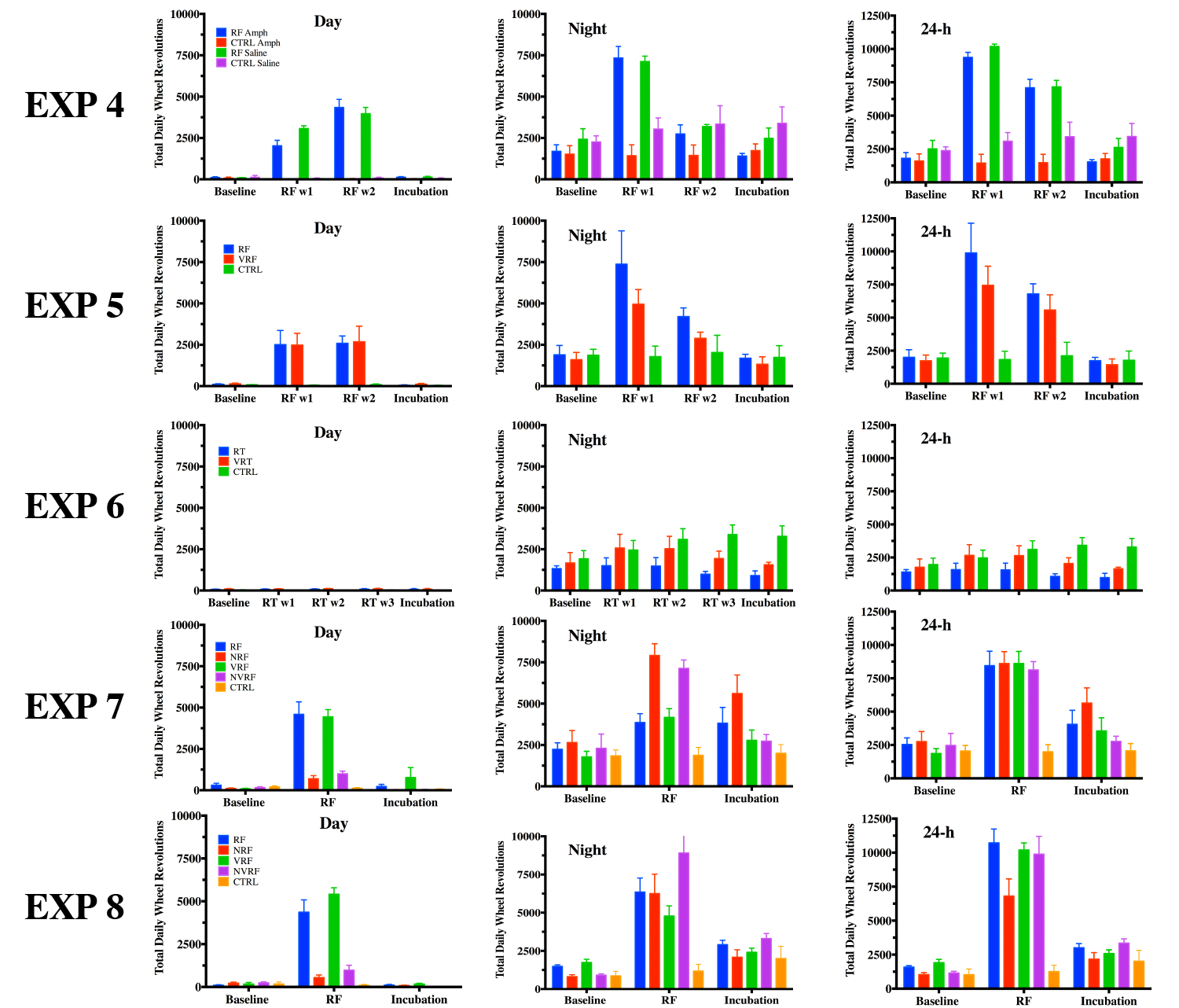

Supplement: S2 Fig — To see whether the total amount of locomotor activity across stages of the experiment differed between feeding groups, total daily wheel revolutions for each rat were summed and averaged for each stage of the experiment, in Exp's 4–8. Group averages of total daily wheel revolutions (+/- SEM) show the amount of activity that occurred in the day, night or over 24-h. (TIF) [file pone.0174113.s002.tif]

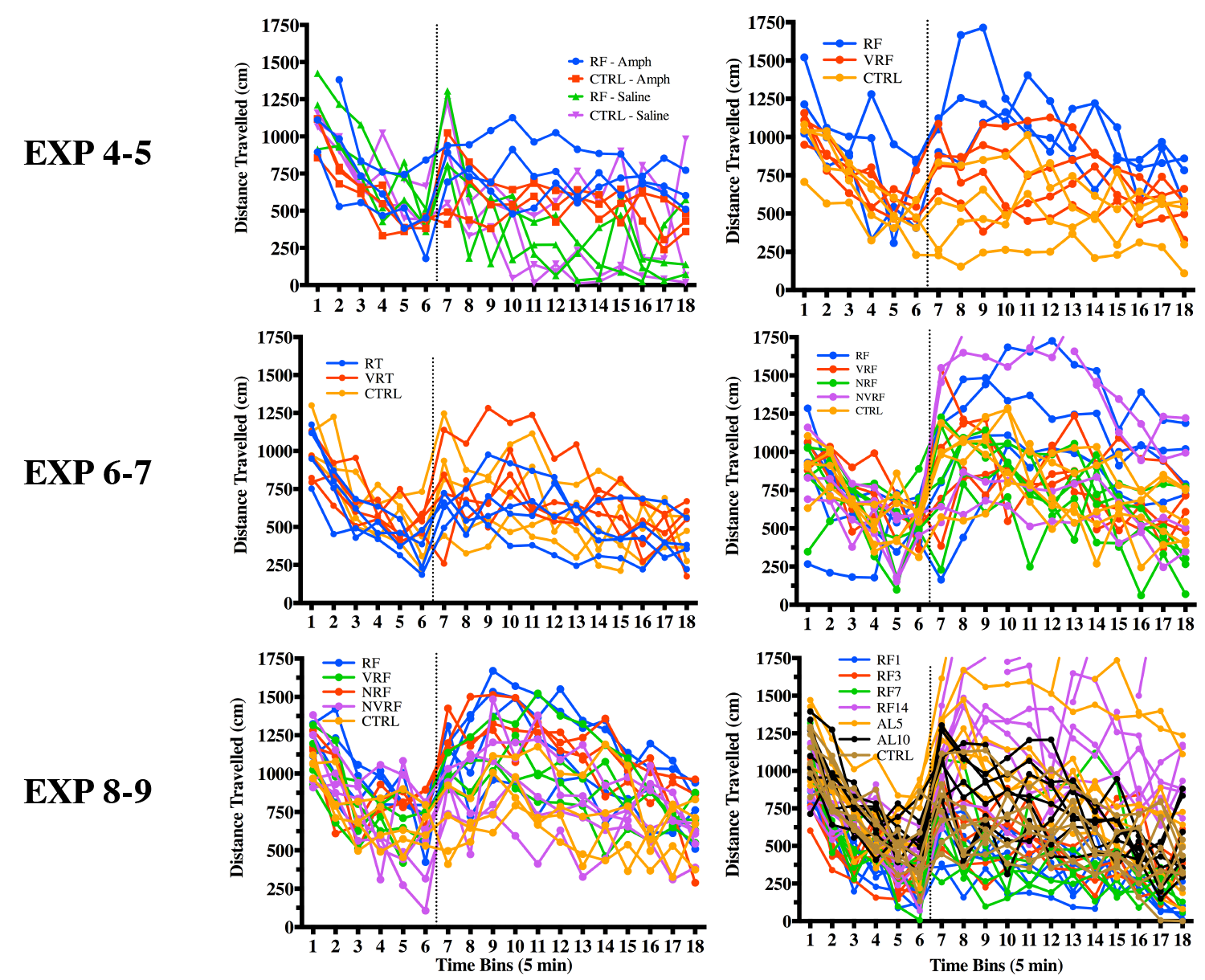

Supplement: S3 Fig — Distance travelled to an acute injection of AMPH, plotted in 5-min time bins +/- SEM for feeding groups in experiments 4–9, respectively. Dotted line indicates injection time. (TIF) [file pone.0174113.s003.tif]
